# Why prospective registration of systematic reviews makes sense

**DOI:** 10.1186/2046-4053-1-7

**Published:** 2012-02-09

**Authors:** Lesley Stewart, David Moher, Paul Shekelle

**Affiliations:** 1 Centre for Reviews and Dissemination (CRD), University of York, Heslington, York, UK, YO10 5DD; 2 Ottawa Hospital Research Institute, Clinical Epidemiology Program, 501 Smyth Road, Ottawa, ON K1H 8L6, Canada; 3 Rand Corporation, Santa Monica, CA 90401-3208, USA

## Abstract

Prospective registration of systematic reviews promotes transparency, helps reduce potential for bias and serves to avoid unintended duplication of reviews. Registration offers advantages to many stakeholders in return for modest additional effort from the researchers registering their reviews.

## Introduction

In this launch issue of *Systematic Reviews*, we publish a series of linked articles about prospective registration of systematic reviews. This is a topic which has received increasing attention in recent years, notably in the 2009 PRISMA guidelines for preferred reporting items for systematic reviews and meta-analyses [1], and the subsequent development [2] and implementation [3] of PROSPERO, an international prospective register of systematic reviews which is described in this issue [4].

We have devoted a substantial part of the first issue of *Systematic Reviews *to this topic because we believe that prospective registration of systematic reviews can play an important part in helping ensure the integrity of the evidence base upon which health policy and treatment decisions are made. A single point of access to information about ongoing reviews should also help avoid the unintended duplication of reviews and wasting of resources, a consideration that is more important than ever in the current economic climate.

### Avoiding bias in the conduct and reporting of systematic reviews

Given that systematic reviews are widely promoted as providing best evidence to inform decision-making, we believe that there is an associated responsibility to promote and encourage best methods and practice to ensure that systematic reviews are as transparent, robust and free from bias as possible.

A key feature of a high-quality systematic review is the development of a protocol that sets out the main objectives, key design features and planned analyses for the review [5-7]. A protocol written in advance of the review should ensure that the review methods are transparent and reproducible, and adherence to this prespecified research plan should help avoid bias.

Although protocol development is integral to systematic reviews carried out or funded by many organizations, authors of only about half of all reviews report having worked on the basis of a protocol [8]. Until the development of PROSPERO, there was no place to register protocol information that is open to all.

Detecting and mitigating bias in included trials and other studies is central to systematic review methods. Yet, the same pressures to be selective in the conduct, reporting and publication are also likely to apply to systematic reviews.

It seems obvious that changes in emphasis that occur between protocols and completed reviews, such as adding or removing outcomes or changing statuses between primary and secondary outcomes, have the potential to bias review findings. In a 2002 study comparing 47 completed Cochrane reviews to their published protocols, the authors found that 91.5% of the reviews contained major changes, many of which pertained to the methods and selected outcomes [9]. In a more recent study, over one-fifth of 64 Cochrane reviews examined over 9 months were found to contain a change in at least 1 outcome from that specified in the published protocol [10]. Discrepant outcomes added or upgraded from secondary to primary at the review stage were more likely to be significant than those outcomes that had not changed. This clearly has the potential to bias the report in favour of the new intervention.

Registration can help guard against outcome and other reporting biases by maintaining a permanent public record of the key elements of the planned review, including the inclusion criteria and intended outcomes. When the review is published, the final results can be compared with what was intended at registration. Readers can then decide whether any discrepancies are likely to have introduced bias. This parallels prospective registration of clinical trials, which has afforded researchers the opportunity to examine, and readers the ability to interpret, the impact of selective reporting in clinical trials.

As with clinical trial registration, there is a need to register reviews prospectively at a point in time when the review protocol has been completed, but ideally before screening for eligibility has begun. This timing will reduce the opportunity for conscious or subconscious manipulation of inclusion criteria to include certain studies to mould a review to reach a desired conclusion. Where reviews are carried out to a short timeline and strict deadlines, registering a review between the completion of the protocol and the start of screening may be challenging, and we accept that for such reviews the initial steps may not always be followed in a strictly sequential manner. For example, screening may start while the wording of the protocol is being finalised or while the authors are awaiting final approval of the protocol from a funder. Pragmatically, this can be tackled by interpreting *completed *to mean when the main elements of the review design have been agreed upon. We argue that the inclusion and exclusion criteria must be agreed upon before screening starts and that the data fields required for registration in PROSPERO are likely to be stable at this point. Thus registration can be done and screening started while the full protocol is being finalised. Any subsequent protocol changes that affect the registration record can be captured in a registration update. In such cases, registration may be advantageous by providing an opportunity to publicly record the main eligibility criteria before screening starts, even if the full protocol has yet to be formally approved and/or published. PROSPERO allows some flexibility regarding the timing of registration and, importantly, captures the stage of the review at which the registration takes place.

It is sometimes necessary and legitimate to make changes from what was planned in the protocol [1]. If an outcome is dropped from a review because no or few included studies reported it, rather than because of unpopular findings, then this does not imply bias, although one might question why the included studies did not report the outcomes that were designated as most important in the review. It is therefore important to know the reasons why a change has been made. A systematic review registry should therefore capture details of any important protocol changes made after the point of registration including the reasons why changes were necessary. This audit trail information should be available publicly.

A simple means of linking a registration record to subsequent review reports and publications is required. PROSPERO has done this by issuing a unique number to each registered review. If the unique registration number is quoted in review publications, which we recommend, then readers can access the PROSPERO site and use the number to look up the associated registration record.

Together these measures will allow public scrutiny and comparison, enabling discrepancies between published analyses and those planned in the registered review protocol to be more readily identified.

Registration will not prevent cheating. Unscrupulous researchers could carry out repeated reviews and/or syntheses and then retrospectively register only those with the desired findings. PROSPERO openly displays dates of registration, amendment and publication, which should provide some deterrence. For example, suspicion would be aroused should a review be registered 1 week and published the next. This does not in itself prevent overt misuse, but we note that falsification of dates would be a deliberate act of scientific misconduct with potentially serious and damaging consequences for the authors if revealed.

### Avoiding unintended duplication

When adopted widely, prospective registration should prove helpful in avoiding unplanned duplication of effort. Systematic reviews are generally time-consuming and costly to carry out, yet often duplicate or similar reviews' are undertaken. Registration should allow those planning reviews to check whether any reviews already in the 'pipeline' address their topic of interest. They can then decide whether to proceed to commission or undertake such a review. Although there may sometimes be good reasons for repeating a review, as three major commissioners of systematic reviews report in this issue [11-13], avoiding unintended duplication is important in ensuring that finite research funds can be used effectively and efficiently.

Given these advantages, in the United Kingdom, the National Institute for Health Research (NIHR) has made registration in PROSPERO compulsory for those systematic reviews that receive NIHR funding and fall within the register's scope. A similar policy is recommended by the Canadian Institutes of Health Research (CIHR).

### Who benefits from registration

Registration allows **researchers **to comply with PRISMA [1] and provides a permanent public record of their planned methods. It may also help raise awareness of their review. Use of the unique registration number may be useful in helping track subsequent use or citation of the review to monitor its impact. Providing a facility with which to link to unpublished reports or grey literature may be helpful, and the registration process may serve to encourage reviewers to publish their findings. Registration of systematic reviews therefore also provides an important resource for those researchers undertaking overviews.

**Commissioning and funding organisations **can utilise PROSPERO to identify ongoing and unpublished reviews to help them avoid unplanned duplication and waste of financial resources [11-13].

**Guideline developers **could use information about forthcoming reviews to assist in the planning and timing of guideline development. For example, they may wish to time guideline development to coincide with completion of relevant ongoing reviews or to contact researchers to request early access to review findings. It may also provide an opportunity for collaboration. The current chair of the Guideline International Development Network describes prospective registration as providing "*an excellent basis from which to seek alignment and create opportunities for including systematic reviewers as members of the guideline development group*" [emphasis added] [14]

**Journal editors **should welcome registration as a safeguard against reporting biases and as a tool to augment peer review.

**Peer reviewers **will be able to link a manuscript to the corresponding registration record (and, where available, through to the full protocol). The registration record and/or the protocol may provide important additional information about, and clarity regarding, the methods that are absent from the manuscript. It could also speed the process because some issues of clarification that would otherwise be sent back to the authors as questions could be resolved by checking the registration record or protocol, thereby circumventing a round of question-and-response between reviewers, journal editors and authors.

Undoubtedly, capturing information about how people design and conduct their reviews will be of interest to **methodologists**, providing an opportunity for observation and research, which may then feed into reviewer education and further methodological development.

Given that an underlying aim of registration is to help ensure that health and social care decisions are informed by good-quality systematic review evidence, registration is also in the **public **interest. Providing open access information about ongoing systematic reviews and encouraging transparency in the systematic review process resonates with the prevailing public mood for openness and disclosure. As many systematic reviews are funded by the public purse, helping to avoid wasting money on unintended duplication should also be welcomed.

### Why registration makes sense

Registration in PROSPERO requires provision of 22 data items, with the option to provide details of a further 18 items, and generally takes around 30 minutes to complete. This does not seem to be an onerous burden on researchers.

The editors of *Systematic Reviews *believe that prospective registration of systematic reviews is an important development that will play a role in promoting transparency and avoiding bias that will ultimately serve to improve methodological standards. Helping to avoid unintended duplication of effort will play a part in ensuring that, globally, research funding can be invested wisely and to best effect. We applaud the lead taken by the NIHR and the CIHR in making registration a condition of systematic review funding, and we hope that other commissioning and funding organizations will follow suit. We are pleased that early signs from PROSPERO indicate that researchers are ready and willing to prospectively register their reviews.

### Declared interests

LS is an NIHR Senior Investigator and Director of the Centre for Reviews and Dissemination (CRD), which designed, implemented and manages PROSPERO. DM and LS are members of the International PROSPERO Advisory Group.
